# Costs of the 'Hartslag Limburg' community heart health intervention

**DOI:** 10.1186/1471-2458-6-51

**Published:** 2006-03-02

**Authors:** Emma T Ronckers, Wim Groot, Mieke Steenbakkers, Erik Ruland, Andre Ament

**Affiliations:** 1 Department of Health Organisation, Policy and Economics, Maastricht University, Maastricht, The Netherlands; 2 Department of Public Health, Regional Public Health Institute Maastricht, Maastricht, The Netherlands

## Abstract

**Background:**

Little is known about the costs of community programmes to prevent cardiovascular diseases. The present study calculated the economic costs of all interventions within a Dutch community programme called Hartslag Limburg, in such a way as to facilitate generalisation to other countries. It also calculated the difference between the economic costs and the costs incurred by the coordinating institution.

**Methods:**

Hartslag Limburg was a large-scale community programme that consisted of many interventions to prevent cardiovascular diseases. The target population consisted of all inhabitants of the region (n = 180.000). Special attention was paid to reach persons with a low socio-economic status.

Costs were calculated using the guidelines for economic evaluation in health care. An overview of the material and staffing input involved was drawn up for every single intervention, and volume components were attached to each intervention component. These data were gathered during to the implementation of the intervention. Finally, the input was valued, using Dutch price levels for 2004.

**Results:**

The economic costs of the interventions that were implemented within the five-year community programme (n = 180,000) were calculated to be about €900,000. €555,000 was spent on interventions to change people's exercise patterns, €250,000 on improving nutrition, €50,000 on smoking cessation, and €45,000 on lifestyle in general. The coordinating agency contributed about 10% to the costs of the interventions. Other institutions that were part of the programme's network and external subsidy providers contributed the other 90% of the costs.

**Conclusion:**

The current study calculated the costs of a community programme in a detailed and systematic way, allowing the costs to be easily adapted to other countries and regions. The study further showed that the difference between economic costs and the costs incurred by the coordinating agency can be very large. Cost sharing was facilitated by the unique approach used in the Hartslag Limburg programme.

## Background

Many western countries have a very high prevalence of cardiovascular diseases (CVD) [1]. In the Netherlands, more than a million persons suffer from CVD [2], corresponding to 8% of the adult population. Because of their high prevalence, CVD lead to high costs. The 1999 medical costs for cardiovascular diseases in the Netherlands, were €3.6 billion [3], corresponding to more than 10% of the total Dutch health care costs.

Reducing risk factors for CVD might reduce the burden that they impose on public health and on the health care budget. There are many ways in which this can be achieved. A promising method to reduce the prevalence of CVD is the implementation of community programmes.

While there is a considerable body of evidence on the effectiveness of community programmes to prevent CVD [4-6], little is known about the costs of these complex interventions. There have been some attempts to calculate the costs of community interventions [7-16], but the studies performed so far have lacked two essential elements, namely (1) costs are not calculated according to guidelines for economic evaluation research and (2) costs are presented at an aggregated level.

It is important that the costs are calculated according to guidelines because previous research has shown that considerable bias may occur in the costs when guidelines are ignored [17-19]. Only if the costs are calculated according to economic guidelines and in a uniform way can they be combined with data on the effectiveness of a programme and used for medical decision making [20,21].

The value of presenting costs of community programmes in a detailed overview instead of presenting the costs at an aggregated level is twofold. First, providing insight into the costs of all individual interventions in a community programme facilitates budgeting for anyone who might want to implement such a programme. The combinations of interventions to change lifestyle that are used in community programmes are different for each programme and it is thus important to know which specific interventions are used, how they are implemented and what their individual costs are. The second advantage of presenting detailed costs instead of only final costs is that the latter may give rise to problems when generalising the costs to other regions and countries, because local factors are likely to affect the costs of a programme. Presenting detailed costs avoids these disadvantages of aggregated presentation.

The present article reports on the costs of the community programme included in the Hartslag Limburg campaign, which aimed at preventing cardiovascular diseases.

The aim of the study was to provide detailed information about costs of interventions within a community programme, calculated in accordance with guidelines for economic evaluation and in such a way as to facilitate generalisation to other countries.

## Methods

### Target population

The intervention region consisted of the town of Maastricht (120,000 inhabitants) and four adjacent municipalities (60,000 inhabitants). The majority of the people thus lived in urban areas. 62% of the inhabitants of the intervention region had paid jobs, while 17% of the inhabitants were older than 65 years.

In the intervention region, cardiovascular diseases accounted for 32% of all deaths. The prevalence of the acute heart infarction in the intervention region was 3.7%, which is almost 2% higher than the average prevalence in the Netherlands.

As regards lifestyle, 32% of the inhabitants smoked and almost 50% of the inhabitants in the intervention region did not meet national recommendations for exercise (30 minutes a day for at least 5 days a week).

### Development of the community programme

The community programme in the Hartslag Limburg campaign aims at health promotion among all inhabitants of the intervention region, that is, the town of Maastricht and four adjacent municipalities (n = 180,000). Special attention was paid to inhabitants of four low socio-economic status (LSES) neighbourhoods (n = 20,000), in which 50% of the interventions took place. The study was approved by the Dutch Medical Ethics Committee TNO (reference CO/TW 2599/10049).

The programme is based on a unique design. Before interventions were implemented, a network was formed consisting of large number of participating organisations. Amongst these organisations were the campaigns coordinating agency, which is the Regional Public Health Institute of Maastricht, as well as other health promotion agencies, the local hospital, general practitioners, welfare services and local authorities. This network can be described as the cornerstone of the campaign. Each of the agencies implemented a number of CVD prevention interventions, and also contributed financially. In this way, the network served as the starting point for the implementation of interventions. The network made it possible to implement a multiple intervention strategy and to reach inhabitants of the region in a variety of ways. Both would have been very difficult to achieve if a network had not been created first.

The community-based health promotion within the campaign was implemented through nine local health committees: one in each of the five municipalities and one in each of the four LSES areas. Each committee consisted of employees of the Regional Public Health Institute and of welfare services, civil servants and individuals from the target population themselves. The committees approached individuals and groups within the target populations by means of health promotion projects and activities.

The participation of community members in the local health committees was essential to facilitate the recruitment and participation of inhabitants. This was especially true for the LSES regions, because their residents are difficult to reach with general health promotion activities.

The participation of civil servants was important as they could effect policy changes, and because health promotion activities are partially financed by city councils.

The choices about which interventions to implement came about in a variety of ways. The most occurring were the following: (1) The coordinating agency or one of the other partners proposed an intervention, which was preferably evidence based. Together with one of the local health committees its value was discussed and the best way to implement the intervention was determined. When necessary partners or 'external' institutions were approached to collaborate in the implementation (2) The health committees explored the needs and wishes of the target-population. Based on this information, existing interventions were implemented or new ones were developed. When cooperation of institutions or organisations was needed, these were approached (3) Agencies outside the network contacted the coordinating agency of Hartslag Limburg with a suggested intervention. Together with the coordinating agency or a local health committee, the suggested intervention was evaluated on its merit for Hartslag Limburg and its attainability. In case of a positive attitude towards the intervention, implementation followed.

The similarity between these ways is that they were based on the observed needs within the community and on the expertise of the various implementers about the methods to reach the inhabitants in the region.

### Cost calculation

An adequate cost calculation requires three steps to be taken, namely identification, quantification and valuation [21].

In the identification phase, decisions were made about the aspects of the complex Hartslag Limburg programme that were to be included in the cost analysis and those that were to be excluded.

Starting from this framework, the quantification step addressed the methods used to obtain an overview of the material and personnel resources necessary for the campaign interventions to be successfully implemented.

Finally, the valuation phase applied the pricing methods.

### Identification

Since the present study focused on the costs of the actual interventions, costs related to creating and maintaining the network were excluded from the analysis. The reason for doing so was that the latter costs were dependent on local organisational aspects, which means that no context-independent estimate was possible. Furthermore, the network was not solely used for the CVD prevention programme, but might serve many purposes. Hence, we were unable to estimate which part of the network costs had to be attributed to the community program of Hartslag Limburg.

We focused on calculating the costs of the interventions. Because of the large diversity of interventions, the aim was to calculate the costs of every single intervention separately. Within Hartslag Limburg, about 1000 interventions were implemented. About 800 of these were large-scale or frequently recurring interventions. In addition, there were about 200 small-scale, nonrecurring events. For practical reasons, only a random sample of the small-scale interventions was used to calculate costs according to the guidelines, and the resulting costs were then extrapolated to all other small-scale and non-recurring interventions. We think this is justified, because of the relatively small contribution of these smaller interventions to the total costs of the programme.

Not all the costs related to the interventions were relevant to the present cost calculation. Costs incurred for the *development *of new interventions were irrelevant because they do not have to be incurred again when the intervention is applied elsewhere, nor would future implementers have to pay indirectly for the development costs. Hence, they were excluded from the analysis. Because future programmes might involve developing new interventions, we provide an indication of the development costs within Hartslag Limburg, which are reported separately.

Finally, a cost calculation according to the guidelines implies that all inputs should be included to obtain a reliable overview of the actual costs of a programme. This also means that all input should be given a monetary value. For the present cost calculation this meant that (1) monetary input or input in kind that was received from other institutions (subsidies, sponsors) was included in the cost analysis, and (2) input from volunteers was also included. Although the guidelines recommend to include input from participants in the cost analysis as well, the current analysis did not include time input by participants, due to lack of data.

### Quantification

In the quantification phase, an overview of all the material and staffing input involved (both in kind and in quantity) was drawn up for every single intervention. The overview was based on documentation, like budget statements, registrations of hours worked by staff, plans of action, evaluation reports and accounts. After an intervention had been implemented, we verified whether the actual input was in agreement with the anticipated input. This was done in regular meetings (once every two months) with health education specialists involved in implementing the interventions. This information was used for an accurate estimation of the resources that were necessary for the intervention to be successfully implemented.

### Valuation

In the third and final step, the staff and material inputs were valued, using Dutch price levels for 2004. Interventions were mainly valued using prices.

#### Staff input

In order to obtain reliable estimates of the costs of staff input, and in accordance with guidelines for cost research [20-22], staff input was valued using salaries based on national mean wage levels for each task level, instead of taking staffing costs in terms of hourly wages directly from the programme's records. This made the cost estimation less dependent on the local context and made it more likely that the estimated costs will correspond to the actual costs of future programmes implemented elsewhere.

In valuing staff input, we assumed an efficient use of input. This meant that adjustments had to be made for inefficient use of staff input by the programme due to start-up problems. When identifying staff input, we used the task level necessary to implement an intervention successfully to define the type of staff input and thus the price attached to it.

#### Material input

The prices of the material input were derived from the Hartslag Limburg records.

### Statistical analysis

The study can be characterized as a descriptive analysis, in which all costs that were made during the community program of Hartslag Limburg were calculated on a detailed level.

## Results

From 1999 until 2003, a total of 790 interventions were implemented. These included 21 different types of large-scale or frequently repeated interventions, accounting for 590 interventions over the five-year intervention period. Table 1 presents an overview of these interventions. Of the 590 major interventions, 193 were aimed at dietary change, 361 were related to physical activity and 9 aimed to make people give up smoking (table 1). There were an additional 27 interventions that aimed at a healthier lifestyle in general. In addition to these interventions, 200 small-scale and non-recurring interventions were implemented. Almost 50% of the interventions took place in disadvantaged areas.

The costs of the individual major interventions and the resulting total costs of the Hartslag Limburg community programme are shown in table 2. Additional file 1 presents an overview of the types and quantities of resources necessary to implement the large-scale or frequently recurring interventions, and tables 3 and 4 present the monetary value of the material and staff input.

As table 2 shows, the total expenditures over the five-year period were estimated to be about €900,000. Of this amount, about €86,000 took the form of so-called start-up costs. These are expenditures that are incurred only once to allow an intervention to be implemented. Examples of such costs in the Hartslag Limburg programme are the training costs of various professionals and of the staff of the butcher's shop involved in one of the interventions. Furthermore, €80,000 of these start-up costs was spent on a single intervention, namely the intervention called 'Tasty and Healthy'. There were large differences in the investments made to influence the various behavioural risk factors. The greatest investments were made to improve exercise habits (about €555,000). The smallest amount was spent on interventions to make people give up smoking (€50,000). On improving the dietary pattern €250,000 was spent. Another €45,000 was spent on improving lifestyle in general.

There were also large differences between the costs of individual interventions. Some interventions were very cheap (e.g. lifestyle seminars, the 'nutrition party' and cycle tours), whereas others involved very high costs, such as the interventions called 'Exercise TV', 'Tasty and Healthy' and 'Focus on Heart and Sports'. These three interventions accounted for 45% of the total costs.

Of the total of €900,000 that was spent on the interventions, the coordinating agency, the Regional Public Health Institute, supplied €100,000, while the Netherlands Heart Foundation paid €185,000 and the municipal authorities contributed about €270,000. Other agencies, companies or organisations – including an well-fare agency – contributed the considerable amount of €345,000. This meant that only a fraction of the costs of the interventions was borne directly by the organising agency.

## Discussion

As far as we are aware, this is the first study to present detailed costs of a large-scale community intervention programme, using the necessary material and staff input for every single intervention as a starting point and applying guidelines for economic evaluation.

The costs of the total programme were about €900,000. The 800 large-scale and recurring interventions accounted for 95% of the costs, while the 200 small-scale and non-recurrent interventions accounted for the remaining 5%.

The three most expensive interventions – 'Exercise TV', 'Tasty and Healthy' and 'Focus on Heart and Sports' – accounted for 45% of the costs. Because of the high costs incurred, it would be of great interest to assess the effectiveness of these interventions. However, the large number of interventions performed simultaneously within the Hartslag Limburg campaign makes it impossible to measure the effects of individual interventions.

The present methodology has some major advantages over those used in previous studies in this field, in terms of validity, reliability, generalisability and health promotion practice.

### Validity

The accuracy with which the costs were calculated by using a bottom-up procedure based on necessary resources, instead of the specific resources used in Hartslag Limburg, improved the validity compared to previous studies. Previous attempts to calculate costs of community programmes were based on retrospectively analysing a programme's financial records. This can lead to considerable bias, because community programmes are often endowed with large contributions (monetary or in kind) by other agencies or organisations, which are often not incorporated in the coordinating agency's financial records and as a result are easily overlooked. Input from volunteers, which was included in the present study, is usually not shown in financial records either.

### Reliability

The bottom-up procedure we used ensured that all relevant costs were included in the analysis, making the study more reliable than previous studies. Unlike previous studies, which used a retrospective design, the present cost calculation was made during the implementation of the Hartslag Limburg interventions, decreasing the degree of bias. Another aspect that contributed to the reproducibility of our results is that efforts were made to detach the costs from the local context, for example by using average salaries for particular task levels instead of using the actual salaries paid within the campaign we studied.

### Generalisability

Using average price levels instead of the actual price levels used within the Hartslag Limburg campaign makes the costs applicable to all parts of the Netherlands, and even to other countries with a price level comparable to that in the Netherlands.

Generalisation to other countries is facilitated by the fact that we have provided a detailed overview of the necessary material and staff input. Generalisation is thus a matter of linking local prices to the necessary material and personnel resources (which are not expected to differ much between regions or even between developed countries).

### Added value for health promotion practice

The insights our study has provided into the resources necessary to perform lifestyle interventions facilitate budgeting, allowing costs to be anticipated.

The main shortcoming of our study is that the bottom-up procedure was not consistently used for the 200 small-scale and non-recurring interventions, but that their costs were estimated from the average costs of similar interventions. However, since these 200 small-scale interventions accounted for only 5% of the total costs, the bias resulting from this adjusted cost calculation method is not large.

A practical disadvantage of the present study was that applying a bottom-up methodology to such a complex programme is very time-consuming.

In spite of the fact that the present study paid more attention to the external validity than previous studies, it must be realized that the costs of community programs will never be completely independent of time and place.

As was shown in the Results section, it is possible to share the costs of such interventions among various parties. If such a cost-sharing approach is to be used in future programmes, it is important to form a network of participating organisations. These partners should be well informed about the goals of the project and the advantages for their own organisation, in order to improve cooperation. Effective collaboration can be promoted by the use of written agreements, especially when private organisations are included. The involvement of the partners can be enhanced by informing them about the progress of the programme. Furthermore, the programme should be brought to the attention of the public, to increase the likelihood of attracting external funding. This public attention can for example be achieved by using the media. Within Hartslag Limburg, it was the task of the project leader to create, maintain and expand the network of partners. It is highly recommendable for future programmes to also appoint a person who can execute this task on a full-time basis. To give an indication of the staffing involved: the Hartslag Limburg coordinating agency invested an annual staff input of at least 1.5 FTEs (including a project coordinator, a health educator and a public relations coordinator).

The main question that remains is whether spending money on programmes like Hartslag Limburg is useful. This question can only be answered by relating the costs of the programme to its effects. Schuit et al. (in press) have already analysed the overall effects of the Hartslag Limburg campaign, and their findings are promising: the community part of the campaign seems to have succeeded in reducing the age- and time-related increase in body weight and blood pressure among the community [23].

Whether the effects of a community intervention like Hartslag Limburg will outweigh its costs will be addressed in a forthcoming cost-effectiveness study.

## Conclusion

The costs of the interventions of the community programme of the Hartslag Limburg campaign were calculated to be €900,000. Because the costs of all interventions were calculated separately, using a bottom-up procedure, our results should provide valuable information to health promotion specialists and policy makers. Furthermore, the methodology used improves the validity and reliability compared to previous studies in this field and it makes it easy to generalise the costs to other settings and countries.

This study further shows that the costs do not have to be borne by one agency. Funding, subsidies and sponsoring can achieve that cost sharing. These can be regarded as valuable potential sources of funding. A network is required, however, to successfully implement a large-scale set of interventions like Hartslag Limburg.

Finally, contrary to previous studies, the present study calculated the costs according to guidelines for economic evaluation. This means that costs can easily be related to the effects of the intervention to calculate a cost-effectiveness ratio, which can be used for decision-making at macro-level.

## Competing interests

The author(s) declare that they have no competing interests.

## Authors' contributions

ETR synthesised the analyses and coordinated the writing. WG conceptualised ideas, and helped to interpret findings. MS assisted in acquiring the data. ER coordinated the implementation of the interventions and contributed to data acquisition. AA conceived of the study, conceptualised ideas and supervised all of the study's aspects. All authors helped to interpret findings, reviewed drafts of the manuscript and gave final approval to the version to be published.

## Pre-publication history

The pre-publication history for this paper can be accessed here:



## Supplementary Material

Additional File 1 **Overview of the types and quantities of the resources necessary to implement the major interventions **The file contains a table that list the resources that are necessary to implement the interventions that were carried out within the community programme of Hartslag Limburg. Click here for file

## Figures and Tables

**Table 1:** Description of interventions within the Hartslag Limburg cardiovascular prevention project.

Intervention	Description
**Nutrition**	
Nutrition party	Discussion meeting about healthy food.
Orientation in supermarket	Education about healthy food in a supermarket.
Lifestyle seminar	Seminar about healthy living, focusing on nutrition.
Personal dietary advice	Based on a questionnaire about dietary habits, people receive written personal advice about dietary changes.
Cookery course	A course about healthy cooking.
Debt assistance	Persons with debts are taught to cook a healthy meal on a small budget.
Tasty and Healthy	Health education programme carried out at a butcher's chain, consisting of several components:
	1) Labelling meat with less than 10% fat
	2) Posters and postcards with healthy recipes
	3) Education about healthy meat provided by the butcher's staff.
**Exercise**	
Walking guide	Guide showing 10 walking routes. Distribution via health centres, media etc.
Walking schedule	Guide showing 70 dates of guided walks.
Walking guide + schedule	Guide showing 10 walking routes and 70 guided walks.
Cycling guide	Guide showing cycling routes.
Exercise TV	Daily TV-guided aerobics programme, including information about the health advantages of exercising.
Walks/cycle tours	Organised walks and cycle tours.
Sports clubs	Weekly group sports activities.
Focus on Heart and Sports	Exercise education at schools, combined with introduction course at a sports club.
**Smoking**	
'Congratulations' campaign	Campaign consisting of five components:
	1) 80 commercials, each lasting 34 seconds, on a local radio station;
	2) 80 billboards ;
	3) Posters and postcards in health centres.
	500 posters and 7500 leaflets were placed in 250 health centres;
	4) Information packages about quitting smoking containing
	8 leaflets and an application form for free nicotine patches (300 requests).
	5) Free nicotine patches (50 requests).
Theatre show on smoking	Intervention consisting of 2 components:
	1) A lesson at school about quitting smoking ;
	2) A theatre show.
Stop smoking around kids	1) An information package containing 9 leaflets and a poster was
	sent to all nursery schools and all health centres in the region;
	2) Personal consultation by welfare worker with parents of
	small children;
	3) Four seminars;
	4) Distribution of 10 billboards;
	5) Distribution of 100 t-shirts with the text: 'no smoking around kids'.
Quit smoking campaign	Anti-smoking campaign using billboards, posters and leaflets.
**Lifestyle in general**	
Lifestyle course	Six 3-hour sessions in which health aspects were discussed with teenagers.
Promotion stand	A display stand with health promotion materials.
Newspaper advertisement	Regular one-page advertisement in local newspaper with information about nutrition, smoking or exercise.

**Table 2:** Costs of Hartslag Limburg cardiovascular prevention project

Intervention	Costs per	Number of	Total costs	Start-up	Development
	Intervention (€)	interventions	(€)	Costs (€)	Costs (€)
**Nutrition**					
Nutrition party	399	59	23.541		
Orientation in supermarket	224	63	14.143		
Lifestyle seminar	234	43	10.083		
Personal dietary advice	8.906	1	8.906	4.000	
Cookery course	1.937	16	30.992		
Debt assistance	534	8	4.272	500	4.500
Tasty and Healthy	50.000	1	50.000	80.000	2.700
Small non-recurring events	200	116	23.200		
Total costs/subsidy relating to nutrition			**165.138**	**84.500**	**7.200**
**Exercise**					
Walking guide	3.212	2	6.425		
Walking schedule	7.510	2	15.020		
Walking guide + schedule	15.910	1	15.910		
Cycling guide	7910	1	7.910		
Exercise TV	2.000	120	240.000		
Walks/cycle tours	255	191	48.705		
Sports clubs	4.179	41	171.339		
Focus on Heart and Sports	39.909	1	39.909		
Small non-recurring events	200	51	10.200		
Total costs relating to exercise			**555.148**		
**Smoking**					
'Congratulations' campaign	11.162	2	22.325	1.500	5.000
Theatre show about smoking	575	3	1.725		
'Stop smoking around kids'	6.955	1	6.955		
Quit smoking campaign	8.727	1	8.727		
Small non-recurring events	200	23	4.600		
Total costs relating to smoking			**44.332**	**1.500**	**5.000**
**Lifestyle in general**					
Lifestyle course	1.557	16	24.912		
Promotion stand	300	10	3.000		
Newspaper advertisement	2.160	7	15.120		
Small non-recurring events	200	10	2.000		
Total costs relating to lifestyle			**45.032**		
Total costs of programme			**809.650**	**86.000**	**13.200**

**Table 3:** Overview of the monetary value of the material input.

Resource	value (€/unit)	source
room rent	25*	Unit price within Hartslag
travelling expenses	0.15**	Dutch manual of cost research
printing costs		Unit price within Hartslag
- posters	0,6	
- leaflet/postcard	0,1	
- covering letter	0,1	
- billboard	4	
- nutritional advice		
questionnaire	0.25	
- book with recipes	7	
(dept assistance)		
- walking/cycling guide	0.8	
- walking guide+schedule	1.5	
- walking schedule	1.5	
- book with introductory		
courses by sports clubs	3	
envelope (A4)	0.15	Unit price within Hartslag
envelope (A5)	0.15	Unit price within Hartslag
stamped envelope (A5)	0.55	Unit price within Hartslag
postage (walking schedule,		
walking/cycling guide)	1.5	National rate
postage	0.4	National rate
distribution of billboard	20	Unit price within Hartslag
distribution of nicotine patches	1.5*****	Unit price within Hartslag
designing newspaper ad	500***	quotation by printer's
publication of newspaper ad	1300***	quotation by printer's
tv production and broadcast	160****	quotation by TV station
radio broadcast	1****	quotation by radio station
nicotine patches	20*****	Unit price within Hartslag
food for cookery course	100	Unit price within Hartslag
t-shirt	10	Unit price within Hartslag
billboard	100	Unit price within Hartslag
* €/hour ** €/km *** €/unit		
**** €/minute tv or radio ***** €/box		

**Table 4:** Overview of the monetary value of the staff input.

resource	value (€/hour)	source
dietician	40	national salary scale*
expert on walks	20	national salary scale*
health expert	45	national salary scale*
secretary	20	national salary scale*
welfare worker	70	national salary scale*
sports teacher	40	national salary scale*

*based on average salary
